# Porous Carbon Sponge from White-Rot Fungus *Phanerochaete chrysosporium* for the Removal of Oils and Organic Solvents

**DOI:** 10.3390/ma16020534

**Published:** 2023-01-05

**Authors:** Yue Gong, Lejie Pan, Huahui Yuan, Juncheng Li, Xin Li, Qian Chen, Yue Yuan, Xian Wu, Sheng-Tao Yang

**Affiliations:** 1 Key Laboratory of Pollution Control Chemistry and Environmental Functional Materials for Qinghai-Tibet Plateau of the National Ethnic Affairs Commission, School of Chemistry and Environment, Southwest Minzu University, Chengdu 610041, China; 2 Key Laboratory of General Chemistry of the National Ethnic Affairs Commission, School of Chemistry and Environment, Southwest Minzu University, Chengdu 610041, China

**Keywords:** biomass carbon, carbonization, oil removal, fungi, environmental remediation

## Abstract

Oil leakage incidentally occurs and leads to environmental disasters. Because of their porous and hydrophobic characteristics, graphene sponges are often studied as an oil adsorbent to repair oil spills at sea. Graphene materials are very expensive, and their biological toxicity has been given serious concerns; however, the easier preparation and eco-friendly, biomass-derived porous carbon materials can be used as an alternative to graphene materials. In this study, we prepared a porous carbon sponge (PCS) for oil and organic solvent removal by carbonizing white-rot fungus *Phanerochaete chrysosporium*, a fast-growing microorganism for the production of lignin-degrading enzymes and the environmental remediation. *P. chrysosporium* fungus balls were converted into black PCS by carbonization at high temperatures, where PCS was light (density of 56 g/L), hydrophobic (contact angle of 115°) and porous. According to the results of BET and XPS analysis, the surface area of PCS was 14.43 m^2^/g, and the carbon in PCS is mainly sp^2^ carbon. PCS could adsorb pure oils and organic solvents within seconds. The adsorption capacities of PCS were 20.7 g/g for gasoline, 30.1 g/g for peanut oil, 27.7 g/g for toluene, 18.5 g/g for dodecane, 32.5 g/g for chloroform, 27.1 g/g for tetrahydrofuran, 23.7 g/g for acetone and 13.7 g/g for ethanol. According to the reusability study, there was no obvious capacity loss after recycling up to 10 cycles. Our results indicated that white-rot fungi could be adopted as a cheap carbon resource for oil and organic solvent removal.

## 1. Introduction

Oils and organic solvents are important pollutants nowadays. The accidental discharge of oils inevitably would induce ecological disaster [1,2,3]. Due to the lower density, oils could float on water, thus blocking the air and light, which leads to the deaths of aquatic organisms. In addition, the biotransformation of oils is usually very slow. Great efforts have been dedicated to remediating oil pollution. To circumvent the oil spill events, commonly applied technologies are developed using insulation (booms) and oil gathering (skimmers) [4,5]. Chemical methods usually use dispersants, solidifiers and bio-reducing agents. The in situ burning of the dispersed fuel is also a possibility. Among these approaches, adsorption is a physical method with the advantages of fast remediation, easy operation, the recovery of lost oil, and low environmental impact [6,7,8].

Porous carbon materials, in particular graphene sponges, attract great interest in oil/water separation due to their huge capacities [9,10,11,12]. In 2012, Zhao and coworkers assembled graphene oxide sheets by hydrothermal treatment with thiourea to prepare a graphene sponge. The adsorption capacity of the graphene sponge for diesel oil reached 129 g/g [13]. Bi et al. annealed graphene oxide aerogel to produce a graphene sponge for oil removal, and the capacities were 20–86 g/g [14]. We reported the one-pot hydrothermal preparation of the graphene sponge, and the as-obtained graphene sponge showed high capacities for oils and organic solvents (23–35 g/g) [15]. The adsorption capacity of graphene prepared by the vapor phase reduction method for oil and organic solvent could be improved to 72–224 g/g [16]. The main limit of graphene sponge is the expensive producing cost. Graphene was used to modify the melamine sponge for oil adsorption [17]. Such a cheaper sponge showed a capacity of 111 g/g for heavy oil. Granular activated carbon was prepared to adsorb and degrade the organics in oil recycled from oil sand [18,19]. Cheap alternatives of graphene sponge have highly demanded oil/water separation [6].

Biomass carbon materials could serve as alternatives to graphene sponges in oil removal applications [20,21]. Banana peel and waste paper were adopted to prepare the hybrid aerogel by freezing-cast, freeze-drying and pyrolysis [22]. The hybrid aerogel showed the adsorption capacities of 35–115 g/g for oils and separated various surfactant-stabilized water-in-oil emulsions with high fluxes up to 8550 L/m^2^/h. Shaddock peel was converted into carbon aerogel by hydrothermal carbonization, freezing-drying and post-pyrolysis process [23]. The carbon aerogel had huge adsorption capacities of 23–48 g/g for oils and organic solvents with good recyclability. Superhydrophobic *Enteromorpha*-derived carbon aerogels showed high performance in multi-behavioral oil/water separation with a capacity of 140 g/g [24]. Sugarcane residue was a good bioresource for carbon aerogel production [25]. The carbon aerogel had capacities of 17.1–37.7 g/g for organic solvents. Plant fibers could be extracted from the *Platanus orientalis* fruit [26]. After vacuum carbonization, the adsorption capacities of 71.72–172.37 g/g were achieved for oils. Beyond biomass from plants, fungi could be used for the preparation of carbon aerogel, too. The mycelia of fungi allow the formation of a porous structure after carbonization. Yi et al. reported a pilot study on bamboo fungus [27]. The bamboo fungus was freeze-dried, coated with carbon ink and carbonized. The fungus carbon aerogel could adsorb 20–42 g/g of oils. Although fungi are good candidates for this purpose, bamboo fungus as a delicious mushroom is too expensive.

White-rot fungi are microorganisms that produce lignin-degrading enzymes. White-rot fungi grow fast and have been applied in environmental remediation, while the preparation of porous carbon sponge (PCS) by white-rot bacteria and its application in the simultaneous removal of oil and organic solvent are rarely studied [28,29,30,31]. In this study, we utilized the fungus balls of the white-rot fungus *Phanerochaete chrysosporium* as the carbon source to prepare PCS for the removal of oils and organic solvents. Fungus balls were homogenized, lyophilized and carbonized to obtain PCS. The as-obtained PCS was characterized by multiple techniques. The adsorption performance of PCS was evaluated in the pure oils and also in the simulated seawater. The regeneration of PCS after adsorbing oils was achieved by evaporation. The implications of the applications of biomass carbon materials in oil removal are discussed.

## 2. Materials and Methods

### 2.1. Preparation of PCS

*P. chrysosporium* strain (MTCC 787) was purchased from Guangdong Microbiology Culture Center and cultivated following the recommended protocols. The recipe of culture medium and cultivation conditions were described in our previous study [28]. After 14 days of cultivation, the fungus balls were collected by filtration to remove the excess culture medium. The fungus mycelia were homogenized and then lyophilized to obtain the white sponge. The white sponge was carbonized under N_2_ atmosphere (99.99%, with flowrate of 150 mL/min) in a tubular furnace at 600 °C for 2 h at a heating rate of 5 °C/min. The hydrophilic white sponge was converted into black PCS after the carbonization. PCS was characterized by multiple techniques, including scanning electron microscopy (SEM, JSM-7500, JEOL, Tokyo, Japan), X-ray photoelectron spectroscopy (XPS, ESCALAB 250XI, Thermo-Fisher, Waltham, USA), infrared spectroscopy (IR, Magna-IR 750, Nicolet, Madison, WI, USA), contact angle measuring device (JC2000D1, Powereach Co., Shanghai, China), X-ray diffraction (XRD, XD-6, Purkinje General Instrument Co., Beijing, China) and Raman spectroscopy (inVia, Renishaw Co., London, UK).

### 2.2. Removal of Oils and Organic Solvents

To visualize the adsorption performance of PCS, chloroform (2 g) was stained red by Sudanred 5B. The chloroform was placed on a glass plate and added with a piece of PCS. The photographs were taken at time intervals of 16 s. At the end of experiment, the plate was wiped with the PCS to remove the residues completely.

Similarly, the visualization of the removal process was performed in simulated seawater. Gasoline (dyed with Sudan red 5B) was added to simulated seawater. The recipe of simulated sea water was as follows: 25 g of NaCl, 1.14 g of CaCl_2_, 0.7 g of KCl and 1 L of deionized water. PCS floated on the simulated seawater and was driven by a pipet tip to suck the gasoline. The photographs were taken before and after adding PCS at a time interval of 2 min.

Adsorption capacities of PCS for oils and organic solvents were measured by weighting PCS before and after adsorption. A piece of PCS was weighted before use (*m*_0_). Then, it was sunk into oil or organic solvent (peanut oil, gasoline, toluene, dodecane, chloroform, tetrahydrofuran, acetone, ethanol). After 24 h, the PCS was collected and weighed again (*m*). The adsorption capacity of PCS was calculated by (*m − m*_0_)/*m*_0_ (g/g). All data contained three duplicates and were expressed as the mean with the standard deviation (mean ± SD).

### 2.3. Recycling of PCS

The recycling evaluation was performed with dodecane. After full adsorption of dodecane, PCS was dried at 80 °C to evaporate the adsorbed dodecane. The dried PCS was used to adsorb dodecane again for the adsorption capacity measurements following the aforementioned protocol. The adsorption capacities were measured up to 10 cycles.

## 3. Results and Discussion

### 3.1. Characterization of PCS

**Figure 1:**
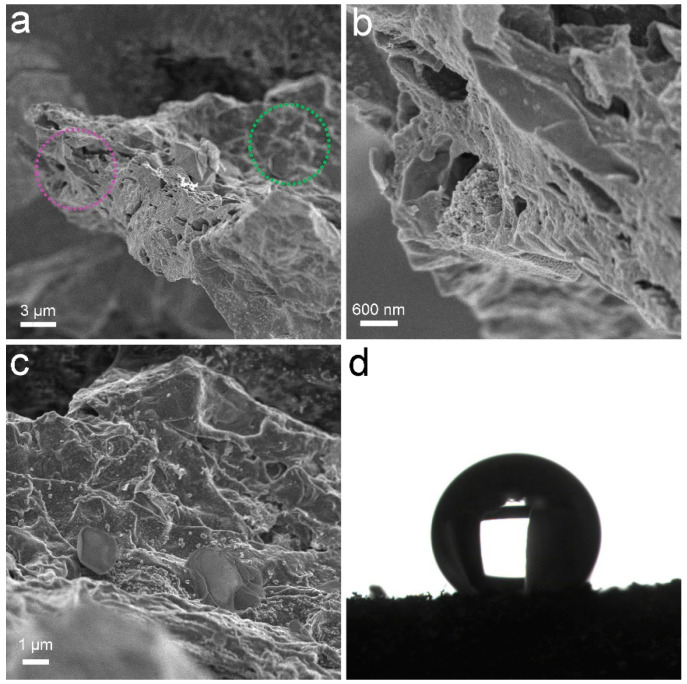
Characterization data of PCS. (**a**–**c**) SEM images; (**b**) Enlargement of the purple cycle; (**c**) Enlargement of the green cycle; (**d**) Contact angle.

**Figure 2:**
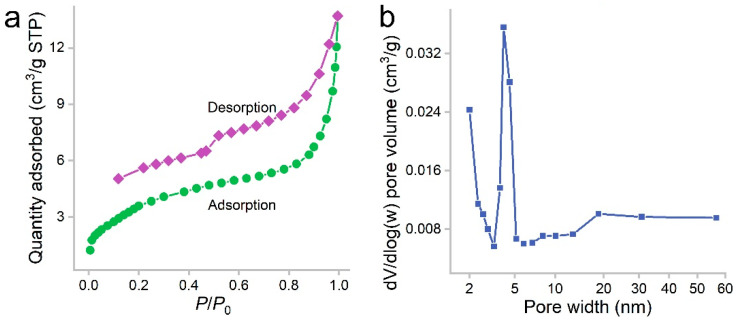
Nitrogen adsorption/desorption isotherms (**a**) and pore size distribution (**b**) of PCS.

PCS was a black sponge. Under SEM, the pores of PCS could be recognized (Figure 1). The surface of PCS seemed tight and flat. The pores were found at the edge of PCS. The presence of pores was important for the adsorption to accommodate oils and organic solvents. These pores were too big to be detectable in N_2_ adsorption/desorption measurements (Figure 2). The contact angle of PCS was 115°, indicating that the hydrophilicity of PCS was moderate. The hydrophilicity was crucial for the adsorption of oils and organic solvents. The N_2_ adsorption/desorption isotherm curve followed type IV, suggesting the adsorption hysteresis nature of N_2_ on PCS. The surface area of PCS measured by the BET method was 14.43 m^2^/g (Figure 2a). The pore width was distributed mostly at 4 nm (Figure 2b) and the pore volume was 0.022384 cm^3^/g.

**Figure 3:**
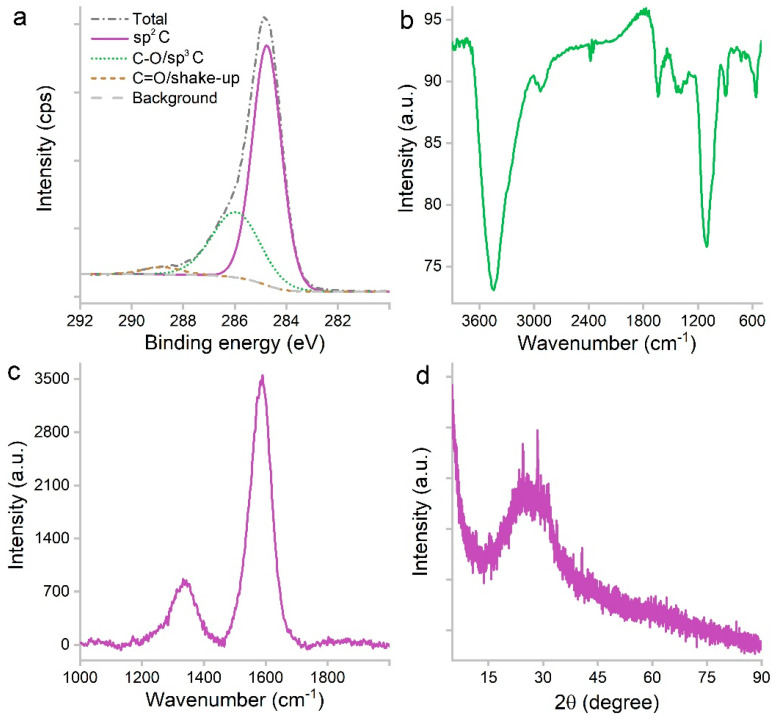
C1s XPS (**a**), IR (**b**), Raman (**c**) and XRD (**d**) spectra of PCS.

The efficient carbonization was indicated by XPS analysis (Figure 3a). There were 79.77% of C, 17.1% of O and 3.13% of N in atomic ratios. The majority of C atoms were in the form of sp^2^ carbon (66.9%) based on the C1s spectrum. The other two components were C-O/sp^3^ carbon (30.43%) and C=O/shake-up signal (2.67%). The oxygen-containing groups were reflected in the IR spectrum (Figure 3b), including 3442 cm^−1^ for -OH/-COOH and 1101 cm^−1^ for C-O. The sp^2^ carbon was identified at 1637 cm^−1^. The small peak at 2931 cm^−1^ was attributed to the remnant hydrocarbon -CH_2_-/-CH_3_. There was no signal at 1720 cm^−1^, suggesting the lack of C=O groups. The lack of negatively charged carboxyl groups explained the hydrophilicity. The sp^2^ carbon was also indicated by the G band (1590 cm^−1^) of the Raman spectrum (Figure 3c). The sp^3^ carbon was indicated by the D band (1340 cm^−1^) as defects. The sp^2^ carbon was in the form of graphene sheets as indicated by the XRD (Figure 3d) band between 13.7–37.8°. The intensity was very low, and the peak was centered at 24.3°, which refers to the 002 crystal face of graphite.

### 3.2. Adsorption of Oils and Organic Solvents by PCS

**Figure 4:**
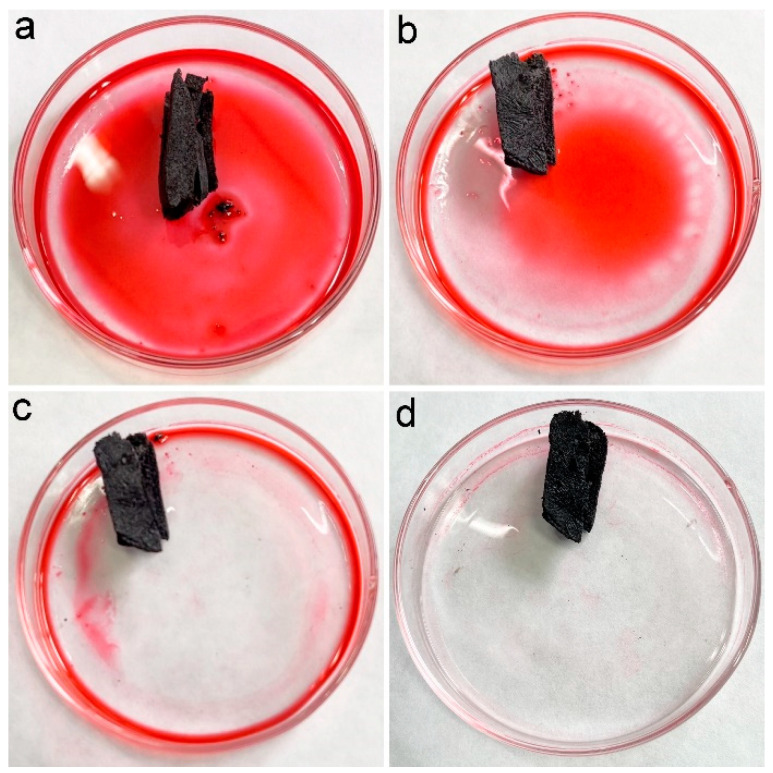
Removal of chloroform (dyed with Sudan red 5B) by PCS. (**a**) chloroform (dyed with Sudan red 5B) before PCS addition (0 s), (**b**) PCS removes chloroform (dyed with Sudan red 5B) for 16 s, (**c**) PCS removes chloroform (dyed with Sudan red 5B) for 32 s, (**d**) PCS removes chloroform (dyed with Sudan red 5B) for 48 s.

In our previous studies, we suggested the accommodation of oils and organic solvents in hydrophobic pores was the main reason for oil adsorption by porous carbon materials [16]. PCS had a contact angle of 115° and a porous structure; thus, PCS was supposed to adsorb oils and organic solvents efficiently. To visualize the adsorption performance, the chloroform was stained with Sudan red 5B, and a piece of PCS was added to the chloroform. As shown in Figure 4, the chloroform was quickly adsorbed into PCS. Within 1 min, all chloroform was sucked into PCS. The quick adsorption of pure chloroform by PCS suggested that PCS could be used for oils and organic solvents.

**Figure 5:**
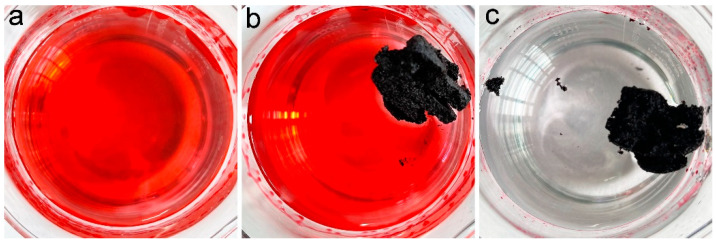
Removal of gasoline (dyed with Sudan red 5B) in simulated seawater by PCS. (**a**) gasoline in simulated seawater (dyed with Sudan red 5B) (0 min), (**b**) PCS starts to remove gasoline (dyed with Sudan red 5B) in simulated seawater (0 min), (**c**) PCS removes gasoline (dyed with Sudan red 5B) in simulated seawater for 2 min.

A more important situation is that oils are leaked into the water environment. Simulated seawater was dumped with gasoline (dyed with Sudan red 5B) (Figure 5). A piece of PCS was added to the beaker, and it floated on the simulated seawater steadily. The PCS was driven by a pipette tip to achieve complete removal within 2 min. After the adsorption, PCS floated on simulated seawater, which could be easily collected.

**Figure 6:**
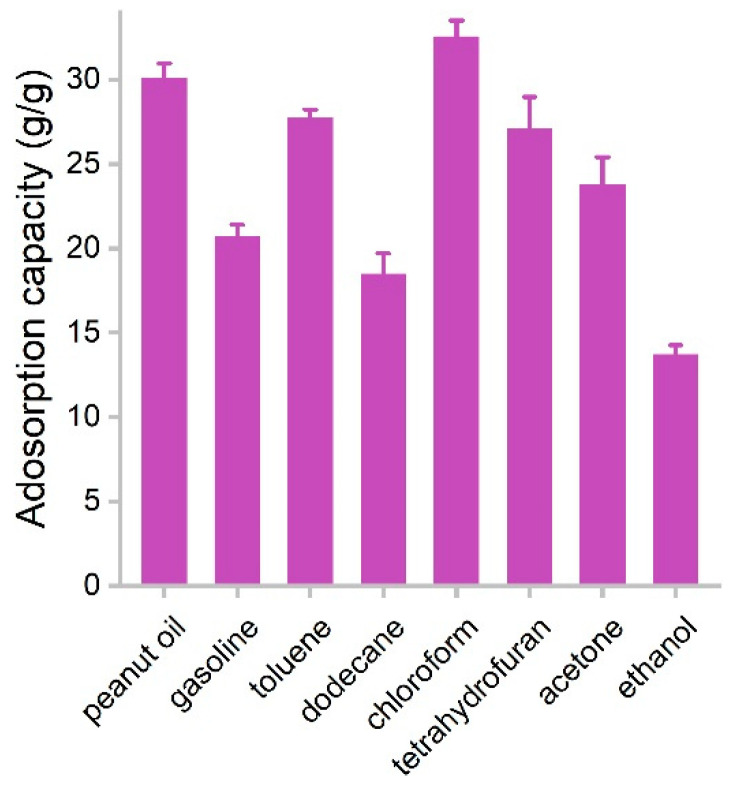
Adsorption capacities of PCS for oils and organic solvents.

The adsorption capacities of PCS for several species of oils and organic solvents were measured in batch experiments. PCS was used to adsorb pure liquids, where the capacities were obtained by weighting PCS before and after the adsorption. The capacities of PCS for oils and organic solvents are presented in Figure 6. The adsorption capacity for ethanol was the lowest, namely 13.7 g/g, which should be due to the higher hydrophilicity of ethanol. The highest adsorption capacity was found in chloroform (32.5 g/g). Rest ranged in the range of 18.5–30.0 g/g. Of particular interest was that PCS adsorbed gasoline efficiently (20.7 g/g), suggesting PCS had the potential for the remediation of oil leakage. The performance of PCS was on the same order of magnitude compared to other biomass carbon materials. For example, carbon aerogels from sugarcane residue had adsorption capacities of 17.1–30.7 g/g for organic solvents [25]. Porous tubular carbon fibers from the fruits of *Platanus orientalis* could adsorb 71.72–172.37 times their own weight [24]. Superhydrophobic *Enteromorpha*-derived carbon aerogels were modified by NH_4_H_2_PO_4_ and showed adsorption capacities of 62–140 g/g for oil and organic solvents [24]. Hybrid aerogels derived from banana peel and waste paper had high oil sorption capacities of 35–115 times their own weight [22]. Carbon aerogel from shaddock peel had 23–48 times its own weight in selectively adsorption oils and organic solvents [23]. Tubelike aerogels from bamboo fungus showed 20–42 g/g capacities for oils and organic solvents [27]. The adsorption capacities of PCS were competitive to graphene sponges from hydrothermal methods [13,14,15]. Compared to the literature results (Table 1) [15,16,25,27,32,33,34,35], the advantages of white-rot fungi in our study included the following. First, white-rot fungi are common fungus species in nature and grow fast. Second, during the growth of white-rot fungi, they could secrete enzymes and might be applied in pollutant remediation. Third, white-rot fungi have mycelia, and the fabrication of porous structures is easy. Due to the aforementioned merits and the high performance, we concluded that white-rot fungi were good precursors of porous carbon sponge for oil and organic solvent removal.

### 3.3. Regeneration of PCS

**Figure 7:**
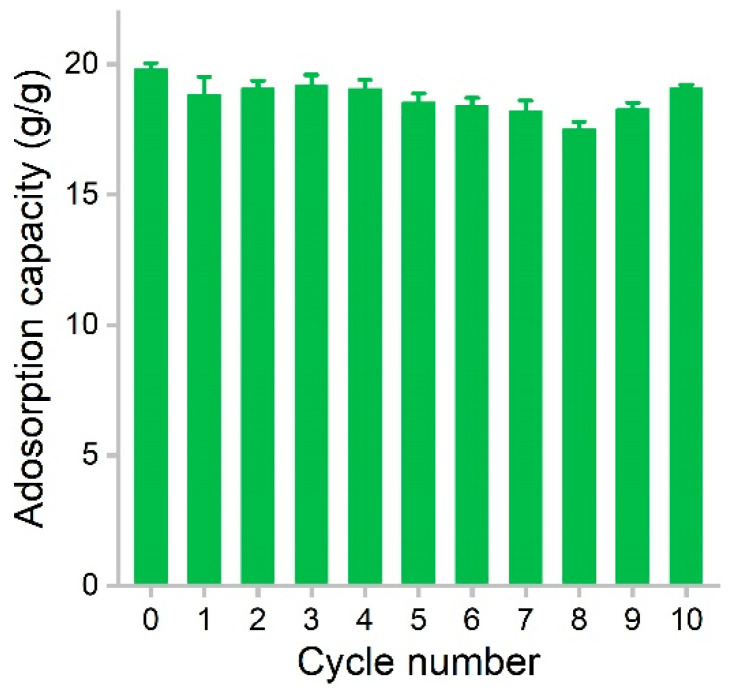
Regeneration of PCS during the removal of dodecane.

The recyclability of adsorbent is a crucial parameter that largely determines the operating cost of the adsorption process. The recyclability of hydrophobic adsorbents depends on the maintenance of the porous structure for the accommodation of oils and organic solvents. In our study, PCS showed very good regeneration performance (Figure 7). Using dodecane as the model solvent, the initial capacity of PCS was 19.8 g/g. After adsorption, the dodecane was evaporated at 80 °C. The surface tension of dodecane was low, so during the drying process, the pores could be retained well. The first regeneration resulted in a capacity of 18.8 g/g. The values varied in the range of 17.5–19.2 g/g during the recycling, and the adsorption capacity was almost always maintained above 90%. At the 10th cycle, the capacity of PCS was 19.1 g/g, equaling 96.5% of the initial value. Compared with graphene sponge, the adsorption capacity is down to 77% of the raw material at 10 cycles [15]. The adsorption capacity was reduced to 78.4% of the raw graphene/polyester staple composite at nine cycles [32]. These results suggested that PCS could be well recycled without obvious capacity loss. In future applications, the reuse of PCS in a real environment should be evaluated.

## 4. Conclusions

In summary, a biomass carbon sponge was prepared by the carbonization of fungus balls, which showed high performance in removing oil and organic solvents. The low density, hydrophobic nature and porous structure enabled the large adsorption capacities of PCS for pure oils and organic solvents within seconds. PCS could float on the simulated seawater to suck oils efficiently. After 10 cycles of regeneration, PCS retained the high capacity. Our results collectively indicated that white-rot fungi were good precursors of porous carbon materials. It is hoped that our results will benefit the ongoing exploration of biomass carbon for oil pollution remediation.

## Figures and Tables

**Table 1:** Comparison of performance between PCS and similar sorbents.

Materials	q_m_ for Oil (g/g)	BET (m^2^/g)	Reference
Porous carbon sponge	20.7	14.43	This study
carbon aerogels	17.1–30.7 g/g	342.34 ± 5.59	[25]
Graphene sponge	29.3	—	[15]
Vapor phase reduction graphene sponge	165	—	[16]
Tubelike aerogels	20–42 g/g	129.61 ± 3.96	[27]
Graphene/polyester staple composite	52	—	[32]
Volume-based magnetic foams	95	89.5	[33]
Magnetic biochar	8.77	48.22	[34]
Coconut oil-modified biochar	5.315	443.81	[35]

## Data Availability

Not applicable.
